# The *ADAMTS18 *gene is responsible for autosomal recessive early onset severe retinal dystrophy

**DOI:** 10.1186/1750-1172-8-16

**Published:** 2013-01-28

**Authors:** Ivana Peluso, Ivan Conte, Francesco Testa, Gopuraja Dharmalingam, Mariateresa Pizzo, Rob WJ Collin, Nicola Meola, Sara Barbato, Margherita Mutarelli, Carmela Ziviello, Anna Maria Barbarulo, Vincenzo Nigro, Mariarosa AB Melone, Francesca Simonelli, Sandro Banfi

**Affiliations:** 1 Telethon Institute of Genetics and Medicine, via Pietro Castellino,111, Naples 80131, Italy; 2 Department of Ophthalmology, Second University of Naples, Naples, Italy; 3 Department of Human Genetics, Nijmegen Centre for Molecular Life Sciences, Radboud University Nijmegen Medical Centre, Nijmegen, The Netherlands; 4 First Neurological Clinic, Department of Clinical and Experimental Medicine and Surgery, Naples, Italy; 5 Medical Genetics, Department of Biochemistry, Biophysics and General Pathology, Second University of Naples; Telethon Institute of Genetics and Medicine, via Pietro Castellino, 111, Naples 80131, Italy; 6 Institute of Biochemistry of Protein (IBP), CNR, Naples, Italy

**Keywords:** Inherited retinal dystrophies, ADAMTS18, Exome, Homozygosity mapping, Medaka fish, Knobloch syndrome

## Abstract

**Background:**

Inherited retinal dystrophies, including Retinitis Pigmentosa and Leber Congenital Amaurosis among others, are a group of genetically heterogeneous disorders that lead to variable degrees of visual deficits. They can be caused by mutations in over 100 genes and there is evidence for the presence of as yet unidentified genes in a significant proportion of patients. We aimed at identifying a novel gene for an autosomal recessive form of early onset severe retinal dystrophy in a patient carrying no previously described mutations in known genes.

**Methods:**

An integrated strategy including homozygosity mapping and whole exome sequencing was used to identify the responsible mutation. Functional tests were performed in the medaka fish (*Oryzias latipes*) model organism to gain further insight into the pathogenic role of the *ADAMTS18* gene in eye and central nervous system (CNS) dysfunction.

**Results:**

This study identified, in the analyzed patient, a homozygous missense mutation in the *ADAMTS18* gene, which was recently linked to Knobloch syndrome, a rare developmental disorder that affects the eye and the occipital skull. *In vivo* gene knockdown performed in medaka fish confirmed both that the mutation has a pathogenic role and that the inactivation of this gene has a deleterious effect on photoreceptor cell function.

**Conclusion:**

This study reveals that mutations in the *ADAMTS18* gene can cause a broad phenotypic spectrum of eye disorders and contribute to shed further light on the complexity of retinal diseases.

## Background

Inherited retinal dystrophies (IRD) are a genetically heterogeneous group of disorders that represent the most frequent causes of genetic blindness in the western world [1,2]. They include Retinitis Pigmentosa (RP) and Leber Congenital Amaurosis (LCA), among the others. RP is the most frequent form of retinal dystrophies with an approximate incidence ranging between 1 in 3000 and 1 in 5000 individuals [1,3,4]. LCA is characterized by a severe visual impairment that starts in the first years of life [5]. Both RP and LCA are generally characterized by a large extent of genetic heterogeneity. Over the last few years, about 230 genes causing inherited retinal diseases have been mapped to chromosomal locations (see RETnet web site: http://www.sph.uth.tmc.edu/RetNet/) and about 190 responsible genes have been identified. It is currently possible to determine the molecular defect underlying IRDs in up to 50% of patients [6], which strongly indicates the existence of additional genes responsible for these conditions.

We recently analyzed a cohort of over 400 Italian patients with autosomal recessive retinal dystrophies, including Retinitis Pigmentosa (ARRP) and LCA ([7] and unpublished data). Patients were screened for the presence of previously described mutations in genes with a known pathogenic role in ARRP and LCA using genotyping microchips based on the allele-specific primer extension (APEX) technique [8]. About 70% of the analyzed patients did not harbor any previously described mutation in known LCA/ARRP genes. We reasoned that this subset of patients could be a valuable resource to identify novel retinal dystrophy genes. Therefore, we selected a subset of the latter patients, preferentially those with some evidence of belonging to consanguineous families, for homozygosity mapping genotyping followed by whole exome sequencing analysis, which has proven to be an effective strategy for the identification of novel autosomal-recessive retinal disease genes [9,10].

## Methods

### Human subjects

We used standard methods to isolate genomic DNA from peripheral blood of the patients and their family members. Informed consent was obtained from all participating patients and families according to the Declaration of Helsinki and the studies were approved by the Research Ethics Committee of the Second University of Naples.

Ophthalmologic examination including best corrected visual acuity using Snellen charts or Teller Acuity Cards, measurement of objective refractive error after cycloplegia, slit-lamp biomicroscopy, dilated fundus examination, bilateral full-field ERGs and optical coherence tomography (OCT) recordings, was carried out as previously described [11].

### SNP genotyping

Genotyping was performed on SNP microarray (GeneChip Genome-Wide Human SNP Array 5.0, Affymetrix, Santa Clara, CA). Array experiments were performed according to protocols provided by the manufacturer. Genotypes were called with the Genotype Console program, ver. 2.1 (Affymetrix) and regions of homozygosity were identified using PLINK [12], with a sliding window of 50 SNPs and allowing 2 heterozygous SNPs (miscalls) and 10 missing SNPs (no calls) per window. Regions containing more than 250 consecutive homozygous SNPs were considered to be significant homozygous regions, on average corresponding to a genomic size of 1 Megabase (Mb).

### Whole exome sequencing

Whole exome enrichment was carried out using the SureSelect All Exon kit v.1 (Agilent Technologies, Santa Clara, CA, USA) according to the manufacturer instructions. Whole exome sequencing was carried out on a SOLiD 3 Plus System (Life Technologies, Carlsbad, CA, USA). Sequencing reads were mapped to the reference genome (UCSC, hg19 build) using the software BioScope v1.3 (Life Technologies, Carlsbad, CA, USA). Single nucleotide variations (SNV) and in-del mutation calling analyses were carried out using the diBayes algorithm with medium stringency settings and the SOLiD Small Indel Fragment Tool, respectively.

### RNA in situ hybridization and immunohistochemistry

RNA in situ hybridization on mouse sections and immunohistochemistry experiments on medaka eyes were performed according to previously published protocols [13,14].

### Medaka stocks and mRNA injections

The Cab-strain of wild type medaka fish were kept and staged as described [15]. A morpholino (Mo; Gene Tools LLC, Oregon, USA) was designed against the ATG initiation codon and the 5^′^ untranslated region of the medaka ortholog of the *ADAMTS18* gene (*olAdamts18*) whereas a control Mo carrying five mismatches (mmMo-Adamts18) was used as a control (see sequences in Additional file 1: Table S2). The specificity and inhibitory efficiencies of Mo-Adamts18 were determined as described in [16]. Mo-Adamts18 was injected at a 90 μM concentration into one blastomere at the two-cell stage. In vitro synthesis of the human wild type *ADAMTS18* mRNAs as well as of the c.T3235 > C mutated form was performed as described [14]. Morpholino and mRNA injections were carried out as previously described [14,17,18].

The light-induced photoreceptor cell degeneration assay was performed as previously described [19]. Medaka embryos were incubated with phenyl thiourea (PTU) to prevent pigmentation [18]. Fish were sacrificed and analyzed after 5 days of constant light.

## Results and discussion

### Clinical features of patient A24

**Figure 1:**
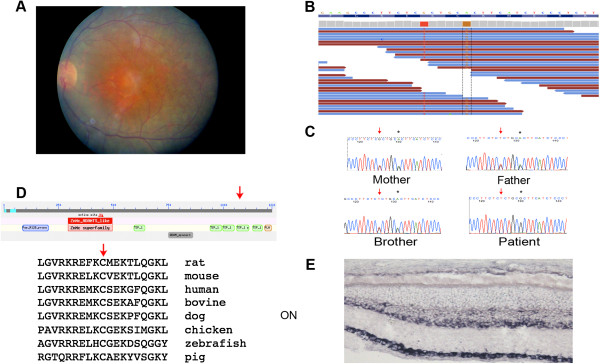
**The *ADAMTS18 *gene is mutated in a patient with Early Onset Severe Retinal Dystrophy.** (**A**) Fundus examination of patient A24 revealed attenuated retinal vessels, macular dystrophy, and diffuse mid-peripheral RPE mottling with myriads of tiny white and black dots. (**B**) Schematic representation, visualized with the Integrative Genomics Viewer (IGV) browser, of the mapped exome sequencing reads around the *ADAMTS18 *c.T3235 > C sequence variant in patient A24. The upper part shows the wild-type sequence and the coverage per base. The c.T3235 > C homozygous variant is shown in brown. Please note that the sequence variant shown in red corresponds to a described SNP (rs35478105). (**C**) Validation by Sanger sequencing of the c.T3235 > C in patient A24 while his parents and his unaffected brother are heterozygous for the variation (red arrows); the rs35478105 SNP is labeled by an asterisk. (**D**) The top diagram shows a schematic representation of the ADAMTS18 protein. The c.T3235 > C (p.C1079R) variant affects a cysteine residue localized within the third TSP type 1 motifs, which is highly conserved across evolution (red asterisk, bottom panel). (**E**) RNA *in situ *hybridization experiment showing that the murine *Adamts18 *is expressed in the adult mouse retina (see text for further details). ON, optic nerve.

Among the selected patients, subject A24, a 30-year old male, displayed a severe early-onset retinal dystrophy accompanied by an autistic disorder. He is the second and last son of two parents that were born in the same small community in Southern Italy. Delivery was at full term without any complication and birth weight was 3750 g. At the age of 2.5, he was diagnosed with autism characterized by emotional indifference and poor/no response to environmental stimuli. A brain CT scan performed at that age was normal. At 2 years of age, the patient started to display exotropia and nystagmus and visual impairment, namely night blindness and reduced peripheral vision. At the age of 5, following a full-field electroretinogram recorded under sedation that showed extinguished rod and cone responses, the patient received a diagnosis of Early-Onset Severe Retinal Dystrophy [20-22]. At the age of 15, he underwent a more detailed ophthalmological evaluation that revealed a hypermetropic refraction (Right Eye: + 2; Left Eye: + 3) and a non-measurable visual acuity due to poor cooperation. Ocular motility testing revealed the presence of exotropia and nystagmus. No signs of fibrillar vitreous condensation were observed by slit lamp analysis. Fundus examination showed attenuated retinal vessels, macular dystrophy, and diffuse mid-peripheral RPE mottling with myriads of tiny white and black dots (Figure 1A). During the follow-up examination at the age of 21, his best corrected visual acuity was 20/1000 and full-field electroretinogram showed extinguished rod and cone responses. At the age of 30, an incipient posterior subcapsular cataract in the right eye was also observed, while all other ocular defects were unchanged. He underwent an OCT (Optical Coherence Tomography) test that demonstrated an absence of foveal depression due to the presence of macular pucker associated with intraretinal cystoid spaces and partial vitreous attachment to the optic disc (data not shown). Complete physical evaluation failed to reveal involvement of other organs and systems.

### Identification of a mutation in the *ADAMTS18* gene in patient A24

We carried out SNP genotyping analysis on a genomic DNA sample from patient A24. This analysis revealed the presence of four large homozygous regions in the genome of the patient, namely on chromosomes 8, 4, 3 and 16 for a total of 15.5 Mb (Additional file 2: Table S1).

We then performed whole exome sequencing analysis on patient A24. Over 65 million sequencing reads, corresponding to about 3.3 Gigabases (Gb) of mappable sequences were obtained using a SOLiD 3 Plus System (Life Technologies, Carlsbad, CA, USA). In total, 86.12% reads could be mapped to the reference genome (UCSC, hg19 build). About 77% of the uniquely mapped reads were aligned on the targeted exome after duplicate reads removal, with 70% of the targeted exons covered at > 20x depth. Overall, we identified 13.388 exonic variants including 6.213 non-synonymous SNVs, 152 small insertions or deletions, 71 stop-gain or stop-loss variants and 40 variants putatively affecting splice sites. Further filtering based on the exclusion of all known dbSNP variants (using dbSNP130) reduced the total number to 760 and the application of an autosomal-recessive model of inheritance for the disease left 110 sequence variants. By limiting the analysis to the regions of homozygosity and by only considering genes with reported evidence of significant expression in the eye [23,24], we were left with only a single homozygous missense variation, namely c.T3235 > C (p.C1079R) in the *ADAMTS18* gene (NM_199355) that is localized to the long arm of chromosome 16 (Figure 1B). Intriguingly, Aldahmesh et al. recently reported a homozygous missense variation [25] in this gene in a Saudi Arabian family with Knobloch syndrome, a rare autosomal recessive developmental disorder that affects the eye and the occipital region of the skull and the brain (On-line Mendelian Inheritance in Man (OMIM) #267750). They therefore proposed *ADAMTS18* as the gene responsible for Knobloch syndrome even if they did not provide any functional evidence in support of their hypothesis [25]. Due to the above-mentioned observations, we regarded this gene as a good candidate for a pathogenic role in the phenotype of patient A24 and therefore selected it for further analysis. ADAMTS18 is a member of the ADAMTS protein family, a family of metalloproteinases similar to the ADAM proteins (A Disintegrin-like And Metalloproteinase), but distinct by the additional presence of ThromboSpondin (TSP) motifs in the C-terminus and the lack of transmembrane domains [26]. The c.T3235 > C mutation causes the substitution of a highly conserved cysteine residue with an arginine in one of the four C-terminal TSP type 1 motifs of the protein (Figure 1D), which are known to be important in modulating ADAMTS-mediated proteolysis [27] and influence protein recognition and matrix localization [28].

Sanger sequencing confirmed that the c.T3235 > C sequence variation was present in homozygosity in patient A24 (Figure 1C). The patient’s parents and his healthy brother were found to be heterozygous carriers of the mutation, which is in line with the autosomal recessive inheritance of the disorder in the family (Figure 1C). The variation was not present in neither the Exome Variant Server (http://evs.gs.washington.edu/EVS/) nor in over 350 Italian control DNA samples. Finally, the c.T3235 > C variation was predicted to be “disease causing” by MutationTaster (http://www.mutationtaster.org/) with high probability and “probably damaging” by Polyphen2 (http://genetics.bwh.harvard.edu/pph2/index.shtml), further supporting its putative pathogenic role.

This is the second report that describes a putative pathogenic role of the *ADAMTS18* gene in human genetic diseases that affect the eye and the central nervous system (CNS). Due to the recent description of another *ADAMTS18* homozygous missense mutation, i.e., the p.S179L, in a family with Knobloch syndrome [25], we carefully revised the clinical history and phenotype of patient A24 to detect a possible overlap with the Knobloch phenotype. However, the lack of any detectable occipital defect as well as the lack of myopia (the patient is actually hypermetropic) and of the classical signs of vitreoretinal degeneration [29] prompted us to exclude the diagnosis of Knobloch syndrome in patient A24 (Figure 1A and data not shown) thus suggesting that the *ADAMTS18* gene is also responsible for non-Knobloch forms of retinal diseases.

To determine whether mutations in *ADAMTS18* are more widely involved in retinal dystrophies, we analyzed 450 unrelated individuals who had ARRP, LCA or autosomal recessive Cone Rod dystrophy and who had significant homozygous regions (see Methods). In 9 families, *ADAMTS18* was located in significantly large homozygous regions: sequence analysis of probands from these families, however, did not reveal the presence of additional mutation in *ADAMTS18*.

### *ADAMTS18* is expressed in the adult eye

*ADAMTS18* was previously reported to be expressed in the developing mouse eye [25]. We aimed at determining whether *ADAMTS18* is also expressed in the adult eye, and mainly in the retina, i.e., the main target of the phenotype present in patient A24. To that purpose, we performed RT-PCR experiments on human retina cDNA (Clontech). As a result, we detected the expected size products (data not shown) using oligonucleotide primers specific for the *ADAMTS18* mRNA sequence (Additional file 1: Table S2) and we confirmed their identity by Sanger sequencing. To define the sites of expression of this gene at the cellular level, we performed RNA *in situ* hybridization experiments on murine adult eye section collected at P60. This experiment revealed the murine *Adamts18* gene to be strongly expressed in the retinal ganglion cell layer (GCL), in the inner part of the inner nuclear layer (INL) and in the retinal pigment epithelium (RPE) (Figure 1E).

### *In vivo* analysis in medaka fish

**Figure 2:**
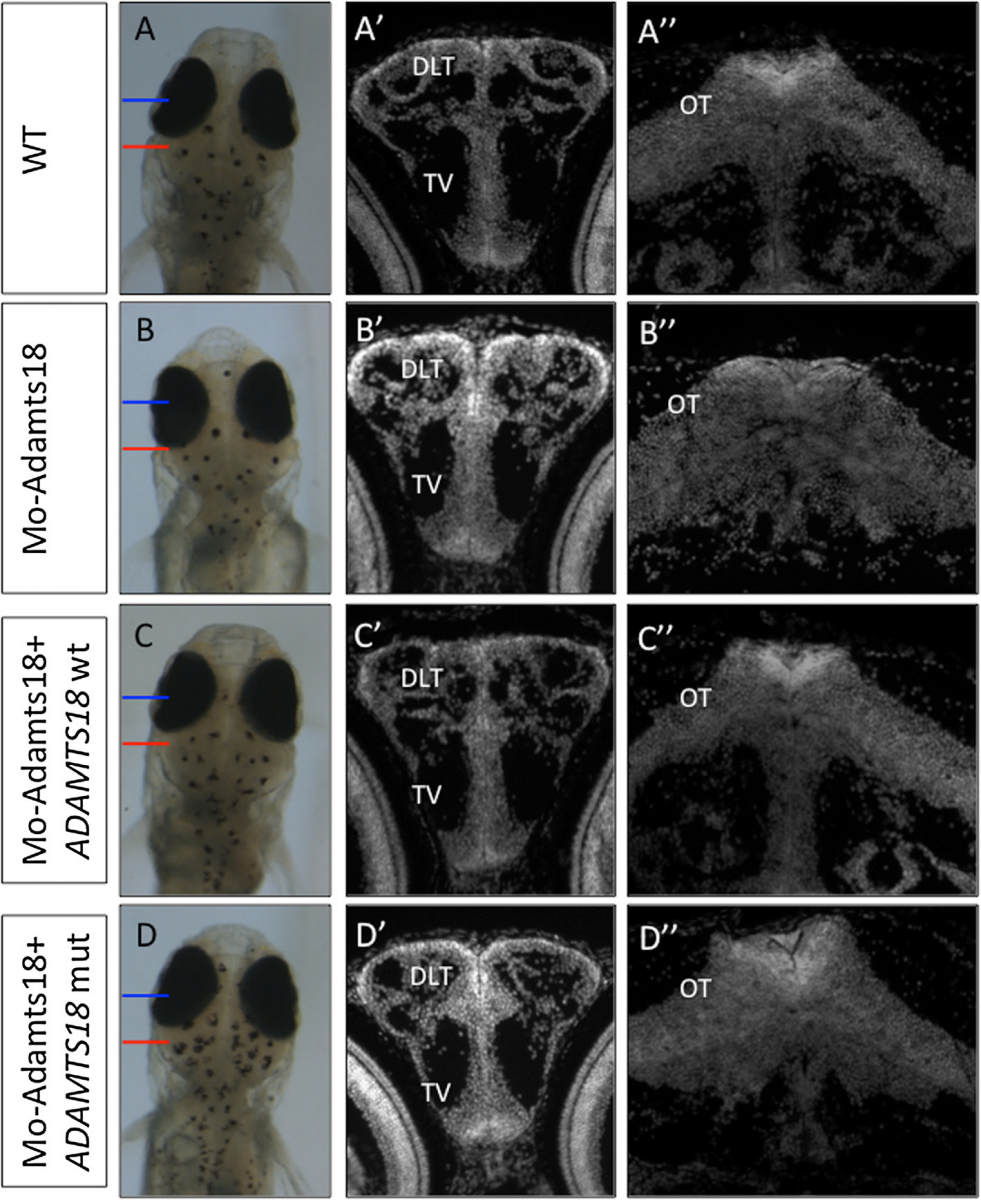
**The c.T3235 > C *ADAMTS18 *mutation has a deleterious effect *in vivo*.** Bright-field stereomicroscopy images of dorsal views of control (**A**), Mo-AdamTS18- (**B**), MO-AdamTS18/ADAMTS18wt- (**C**), and MO-AdamTS18/ADAMTS18mut- (**D**) injected medaka embryos. (A’,A”,B’,B”,C’,C”, D’,D”) Frontal cryostat sections of St40 medaka embryos stained with DAPI (white). Panels A’-D’ are frontal sections through the telencephalon (the section plan is marked by the blue line), whereas panels A”-C” are frontal sections through the mesencephalon (the section plan is marked by the red line). (B’) In Mo-AdamTS18-injected embryos, both the dorsolateral part of the telencephalon (DLT) and the size of the telencephalic ventricles (TV) are altered with respect to control embryos (A’). (B”) Expansion of the optic tectum (OT) in the mesencephalon is present in Mo-AdamTS18-injected embryos. (C’-C”) Wild-type human *ADAMTS18 *mRNA co-injection with Mo-AdamTS18 restores the correct pattern of both telencephalic and mesencephalic tissue. (D’-D”) Co-injection of the human *ADAMTS18 *mRNA carrying the c.T3235 > C mutation with the Mo-AdamTS18 does not rescue the telencephalic and mesencephalic phenotypes.

To further prove the pathogenic effect of the *ADAMTS18* c.T3235 > C variation in patient A24, we carried out an *in vivo* functional test in the *Oryzias latipes* (medaka fish) model system by means of gene knock-down and rescue assays, as already described [17]. We designed a specific morpholino oligonucleotide against the ATG initiation codon and the 5^′^ untranslated region of the medaka ortholog of the *ADAMTS18* gene (*olAdamts18*) (Additional file 1: Table S2) and we injected it into fertilized one-cell embryos. We observed an aberrant CNS phenotype at stage (st)40 in morphant embryos (79 ± 3% of 1,300 injected embryos), particularly in the telencephalic and mesencephalic areas (Figure 2A-B”) of the brain. In particular, we observed an altered morphology of the dorsolateral part of the telencephalon (DLT in Figure 2B’) and a size reduction of the telencephalic ventricles (TV in Figure 2B’). Furthermore, we observed an expansion of the optic tectum in the mesencephalon (OT in Figure 2B”). Interestingly, the areas of the medaka brain affected by the *olAdamts18* knockdown correspond to the most posterior areas of the human brain, which can be altered in Knobloch syndrome patients (OMIM #267750). The above-described aberrant medaka phenotypes were significantly rescued when the *olAdamts18* morpholino was co-injected with the full-length coding human *ADAMTS18* synthetic mRNA (93 ± 2% of 600 injected embryos; Figure 2C-C”) but not with the human mRNA carrying the c.T3235 > C mutation (Figure 2D-D”). These data provide *in vivo* evidence that the c.T3235 > C missense variation in the *ADAMTS18* gene has a pathogenic effect. We believe that the *in vivo* gene knockdown/rescue assays in medaka can be used as a rapid and efficient tool to screen the possible pathogenic role of sequence variants identified in the course of whole exome or whole genome sequencing efforts in patients with genetic diseases, particularly in those characterized by a developmental phenotype.

**Figure 3:**
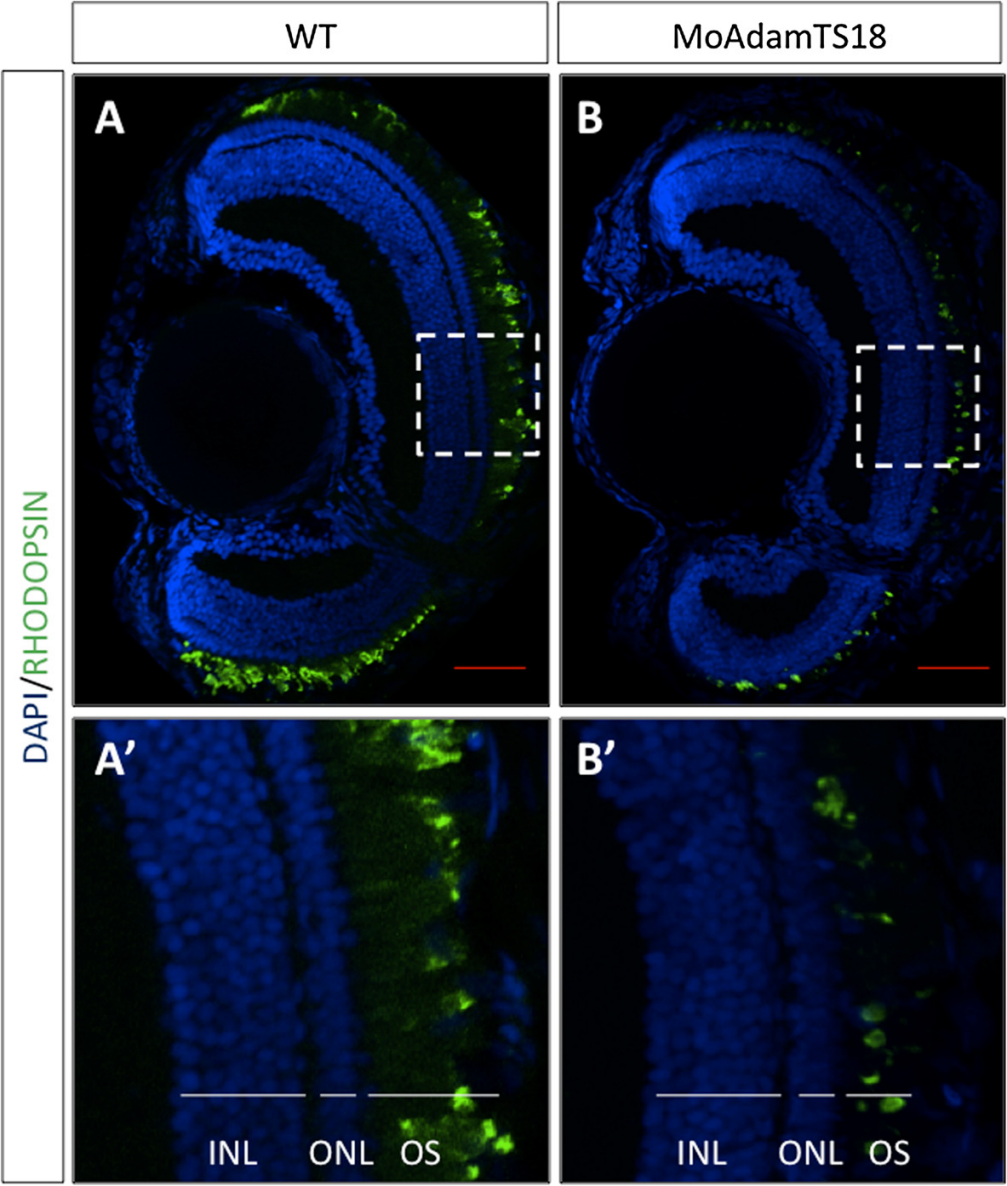
**Exposure to intense light induces a severe retinal degeneration in Mo-AdamTS18-injected medaka fish. **Representative frontal eye sections, immunostained with an anti-Rhodopsin antibody (green), from P5 control (**A**) and Mo-AdamTS18–injected (**B**) medaka fish after 5 days of constant light exposure. Sections are counterstained with DAPI (blue). We observed a significant reduction in both the thickness of the photoreceptor outer segment (OS) and in the intensity of rhodopsin staining in Mo-AdamTS18–injected medaka fish as compared with control animals following constant and intense light exposure. **A’ **and **B’ **are higher magnifications of the areas marked by the white dashed boxes in A and B, respectively. Other abbreviations: ONL, Outer Nuclear Layer; INL, Inner Nuclear Layer. Scale bars, red lines: 20 μm.

We could not detect any gross morphological abnormality of the eye in morphant embryos until st40. Therefore, in order to gain further insight into the involvement of *ADAMTS18* in retinal function in vertebrates, we decided to perform a functional test in medaka fish. In particular, we decided to use the light-induced photoreceptor degeneration model. In teleost fish, including medaka fish, exposure of the retina to intense light is a noninvasive method to elicit the selective degeneration of photoreceptors [30]. We carried out this test in st40 *olAdamts18* knockdown embryos and we analyzed their retinas 5 days later (post-hatching stage, n = 20 eyes). We observed a notable increase of light-induced rod photoreceptor damage in *olAdamts18* knockdown vs. wild type fish, as assessed by immunofluorescence analysis with an anti-Rhodopsin antibody (Figure 3). In particular, we detected a reduction of about 50% of the Rhodopsin-positive retinal areas in *Adamts18*-deficient eyes as compared with wild-type eyes, following light damage. These data strongly indicate that *ADAMTS18* is required for proper photoreceptor function and further corroborate the pathogenic role of this gene in inherited retinal dystrophies. The evidence that this gene does not seem to be expressed in photoreceptors (Figure 1E) is not in contrast with the above findings. *ADAMTS18* is expressed in the RPE, a tissue that plays a pathogenic role in many forms of retinal disorders [31,32].

## Conclusions

We determined, by using a multidisciplinary strategy involving the use of advanced genomic procedures and *in vivo* functional analysis, that mutations in the *ADAMTS18* gene, recently proposed to cause Knobloch syndrome, can also be responsible, although with a relatively low frequency, for early-onset severe retinal dystrophy possibly accompanied by other CNS features, such as autism and neurodevelopmental delay. Importantly, the results of our *ADAMTS18* knockdown experiment in medaka provide for the first time *in vivo* functional support to the pathogenic role of this gene in Knobloch syndrome as well. Our data indicate that different mutations in the *ADAMTS18* can be linked to the pathogenesis of different eye disorders and contribute to shed further light on the molecular mechanisms underlying the complexity of inherited retinal dystrophies.

## Competing interests

All authors have non-financial interests that may be relevant to the submitted work.

## Authors’ contributions

IP, MP and CZ performed the genotyping and sequencing analysis. FT, AMB, MABM and FS recruited the family described herein and collected the clinical data. RWJ, GD and MM performed the statistical interpretation of linkage and sequencing analyses. analyses. NM performed the gene expression analysis. The members of the European Retinal Disease Consortium involved in this study contributed to the mutation analysis on a large dataset of retinal dystrophy patients. Sara B and IC carried out the *in vivo* functional studies. IP, IC, VN, FS and SB oversaw all aspects of the research. SB initiated, planned and coordinated the study. IP, IC, FS and SB wrote the manuscript. All authors read, edited and approved the final version of the manuscript.

## Supplementary Material

Additional file 1: Table S2 Oligonucleotide primers used in this work. Click here for file

Additional file 2: Table S1 List of large homozygosity regions identified in patient A24. Click here for file
