# Supplementary material for: The ADAMTS18 gene is responsible for autosomal recessive early onset severe retinal dystrophy
Source: Orphanet J Rare Dis. 2013 Jan 28;8:16. doi: 10.1186/1750-1172-8-16 (PMC3568033; doi:10.1186/1750-1172-8-16)
Supplement: Additional file 1: Table S2 — Oligonucleotide primers used in this work. [file 1750-1172-8-16-S1.doc]

**Supplementary table 2.** Oligonucleotide primers used in this work

| *Oligo name* | *Sequence* | *Species* | *Purpose* |
| --- | --- | --- | --- |
| ADAMTS18_S1f | TGTGATCCTGGCAGTCTTCA | human | Amplification of the region surrounding the c.T3235C mutation for Sanger sequencing analysis |
| ADAMTS18_S1r | CACGGCAATGAATACCATCC |
| m_Adamts18_S1f | ATAAGGGCCTGTACCTCAGTCA | mouse | Generation of the template for RNA in situ hybridization analysis of the mouse *Adamts18* gene |
| m_Adamts18_S1r | GTGCTCACTGGACAGAGGGAG |
| MoAdamts18 | CAAGCTTGCGCCTACCTCCTCAAAG | medaka | Morpholino to knockdown the medaka *Adamts18* gene |
| ADAMTS18_RT-5’F | CGCACCTGACCATGGAGT | human | Amplification of human retinal cDNA for RT-PCR analysis |
| ADAMTS18_RT-5’R | AACTTTACGCACTGGCCTTG |  |
| ADAMTS18_RT-3’F | CAAGGCCAGTGCGTAAAGTT |  |  |
| ADAMTS18_RT-3’R | GCATGACTTGCAGCATTGTT |  |  |
