# Supplementary material for: The ADAMTS18 gene is responsible for autosomal recessive early onset severe retinal dystrophy
Source: Orphanet J Rare Dis. 2013 Jan 28;8:16. doi: 10.1186/1750-1172-8-16 (PMC3568033; doi:10.1186/1750-1172-8-16)
Supplement: Additional file 2: Table S1 — List of large homozygosity regions identified in patient A24. [file 1750-1172-8-16-S2.docx]

**Supplementary Table 1.** List of large homozygosity regions identified in patient A24

| Chromosome | Genomic coordinates (hg19) |
| --- | --- |
| 3 | 137,250,044-139,407,384 |
| 4 | 175,474,895-179,033,275 |
| 8 | 46,924,211-51,593,888 |
| 16 | 73,822,554-78,950,254 |
